# First Report of Response to Tarlatamab in a Patient With Histologic-Transformed SCLC From *ALK*-Rearranged NSCLC: Case Report

**DOI:** 10.1016/j.jtocrr.2025.100941

**Published:** 2025-12-12

**Authors:** Kaiwen Wang, Ceylan Altintas Taslic, Patricia de Groot, Mitchell A. Parma, Alvaro Guimaraes Paula, Melody Caranto, Komal Shah, Mukulika Bose, Cole Ruoff, Loukia G. Karacosta, Lauren A. Byers, Carl M. Gay, Jianjun Zhang, John V. Heymach, Bingnan Zhang

**Affiliations:** a Division of Pharmacy, The University of Texas MD Anderson Cancer Center, Houston, Texas; b Department of Neuroradiology, The University of Texas MD Anderson Cancer Center, Houston, Texas; c Department of Thoracic Imaging, The University of Texas MD Anderson Cancer Center, Houston, Texas; d Division of Cancer Medicine, The University of Texas MD Anderson Cancer Center, Houston, Texas; e Department of Thoracic, Head and Neck Medical Oncology, The University of Texas MD Anderson Cancer Center, Houston, Texas; f Cancer System Imaging, The University of Texas MD Anderson Cancer Center, Houston, Texas; g Graduate School of Biomedical Science, The University of Texas MD Anderson Cancer Center, Houston, Texas

**Keywords:** Tarlatamab, Small cell transformation, ALK, Lorlatinib, Case report

## Abstract

Small cell transformation has been described as a resistance mechanism to targeted therapy treated in patients with *EGFR*-mutated NSCLC and less often reported with those with other actionable oncogenic alterations, including *ALK*-rearranged NSCLC. Given lack of standard-of-care treatments for patients with actionable oncogenic alteration NSCLC transformed to SCLC, this remains a challenge and unmet need for treating these patients.

Here, we present a case of a patient with *ALK*-rearranged NSCLC with transformation to SCLC, who has progressed on several lines of therapies and successfully treated with tarlatamab to elicit and maintain clinical benefit, including intracranial response.

## Introduction

ALK rearrangement accounts for approximately 4% of targetable oncogenic driver in NSCLC cases.^1^ Multiple generations of *ALK* inhibitors have been developed with alectinib, brigatinib, and lorlatinib found to have favorable survival outcomes compared with the earlier generation *ALK* tyrosine kinase inhibitors (TKIs), such as crizotinib and ceritinib.^2^, ^3^, ^4^ Progression-free survival (PFS) with alectinib was significantly longer compared with crizotinib (34.8 mo versus 10.9 mo, hazard ratio [HR] 0.43, 95% confidence interval [CI]: 0.32–0.58). This also translated to overall survival (OS) benefit with alectinib (not reached versus 57.4 mo, HR 0.67, 95% CI: 0.46–0.98).^2^ Recently, lorlatinib demonstrated significantly better survival outcome and great intracranial response in patients with *ALK*-rearranged NSCLC. Solomon et al.^4^ reported that at 5 years median PFS for lorlatinib was not reached and was 9.1 months with crizotinib (HR 0.19, 95% CI: 0.13–0.27) and median time to intracranial response was not reached (HR 0.06, 95% CI: 0.03–0.12). However, histologic transformation to SCLC remains one of the rare but aggressive resistance mechanisms to *ALK* inhibitors and poses as a challenge in clinical practice to improve patient outcomes.^5^, ^6^, ^7^, ^8^

Treatment of small cell transformation generally involves the use of platinum-etoposide with or without immunotherapy, and outcomes are generally poor.^9^^,^^10^ In addition, SCLC cell surface antigens such as DLL3 have been detected in transformed SCLC.^10^ Tarlatamab, a bispecific T-cell engager that targets DLL3 on tumor cell and CD3 on T cell, is a newly approved treatment option for patients with relapsed extensive-stage SCLC.^11^^,^^12^

In this report, we present a patient case of *ALK*-rearranged lung adenocarcinoma with SCLC transformation after *ALK* TKI treatment and subsequently treated successfully with tarlatamab while continuing lorlatinib.

## Case Presentation

**Figure 1:**
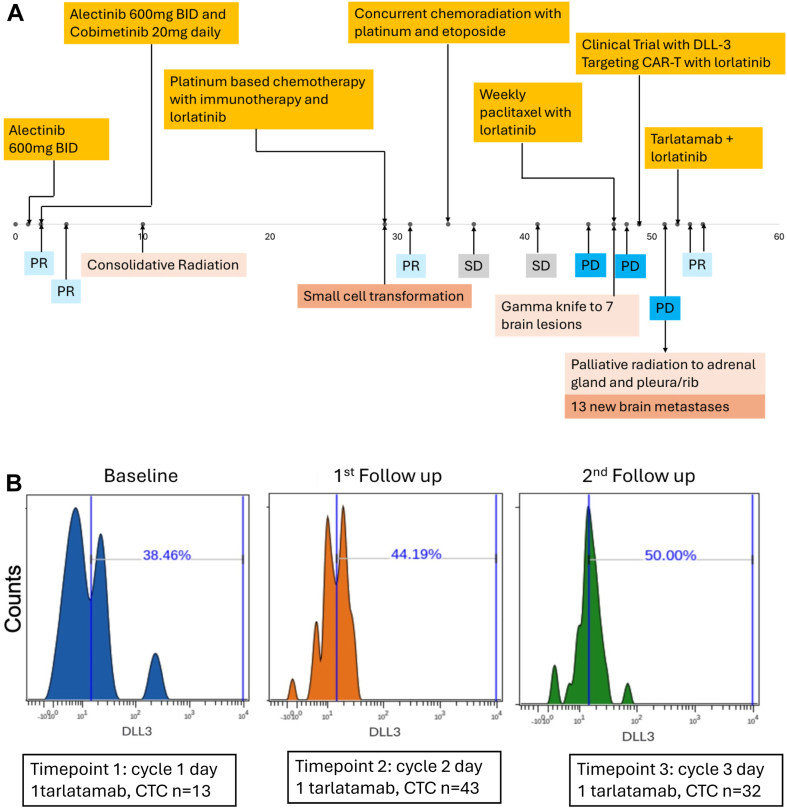
(*A*) Treatment timeline. (*B*) DLL3 protein expression in CTCs detected in longitudinal liquid biopsies from patient. Histograms revealing DLL3 protein expression levels and percentage-positive cells for DLL3 in CTCs detected in three longitudinal liquid biopsies from patient on tarlatamab treatment as assessed by CyTOF. CAR, chimeric antigen receptor; CTC, circulating tumor cells; CyTOF, Cytometry by Time-Of-Flight; PD, progressive disease; PR, partial response; SD, stable disease.

A 42-year-old white male and never smoker presented with cough and was diagnosed with *EML4-ALK* (*E13A20*) fusion-positive lung adenocarcinoma with left lung primary and metastases to the brain, bones, and adrenal gland. Next-generation sequencing (NGS) on tissue revealed *TP53* p.R248W and two *ATM* mutations with tumor mutational burden of 0.8 (Fig. 1*A*). Pathology review and NGS were completed at outside institution with reports available, but no original pathology slides or immunohistochemistry assay images available for review.

The patient was initiated on alectinib 600 mg twice daily with partial response on computed tomography (CT) and magnetic resonance imaging (MRI). He was then enrolled in a clinical trial and received alectinib 600 mg twice daily with cobimetinib 20 mg daily on days 1 to 21 every 28 days and achieved partial response. He received consolidative radiation therapy to his chest 8 months after starting the clinical trial. Then, 16 months after completing radiation, he developed radiologic progression of the primary tumor and new lymph node involvement on positron emission tomography (PET)/CT. Biopsy results demonstrated adenocarcinoma with small cell features. NGS on tissue revealed that *EML4-ALK* fusion and the *TP53* mutation were retained, and *ATM* mutations were lost with an increased tumor mutational burden to 7. He proceeded with receiving platinum-based chemotherapy with immunotherapy and lorlatinib followed by concurrent chemotherapy and radiation to the chest. One year after completing radiation, he developed radiologic progression of bone metastases and new adrenal gland lesion on PET and several new brain lesions on MRI. He received paclitaxel 80 mg/m^2^ weekly with concurrent radiation to progressive bone lesions and gamma knife radiation to seven brain lesions. Subsequently, he enrolled in a DLL3 targeting chimeric antigen receptor (CAR) T-cell clinical trial in an outside institution. Six weeks after receiving DLL3 CAR T-cell therapy, he developed radiologic progression in the left adrenal gland with new pleura and brain metastases. A repeat biopsy confirmed small cell recurrence with no reported adenocarcinoma component in the adrenal gland, and DLL3 expression was reported to be greater than 90% (immunohistochemistry clone SP347, intensity of staining: moderate). NGS on tissue demonstrated new loss-of-function alterations in *RB1* and *PTEN* with retention of *EML4-ALK* fusion and *TP53* mutation. Pathology review and NGS were completed at outside institution with reports available, but no additional pathology material for review. He then received radiation to the adrenal gland and pleura before initiating tarlatamab. Baseline brain MRI before tarlatamab revealed 13 new subcentimeter brain metastases. Intriguingly, he had three longitudinal blood samples banked on tarlatamab treatment under the IRB-approved GEMINI protocol. As preliminary data, we analyzed DLL3-positive circulating tumor cells (CTCs) in his blood samples and found stable percentage of DLL3-positive cells based on established cytometry by time-of-flight (CyTOF) methodology (Fig. 1*B*).^13^

**Figure 2:**
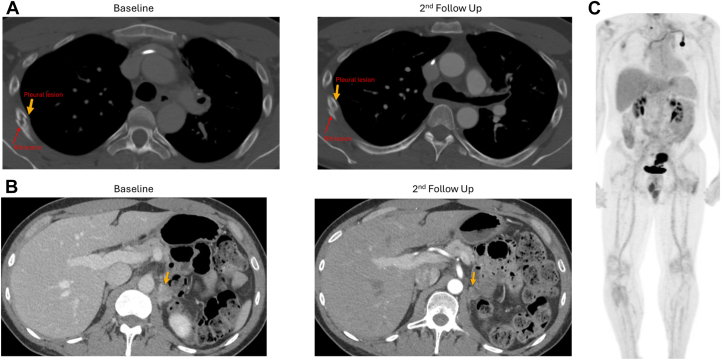
Systemic response to tarlatamab plus lorlatinib. (*A*) Pleural and rib lesion. (*B*) Adrenal lesion. (*C*) First follow-up PET.PET, positron emission tomography.

**Figure 3:**
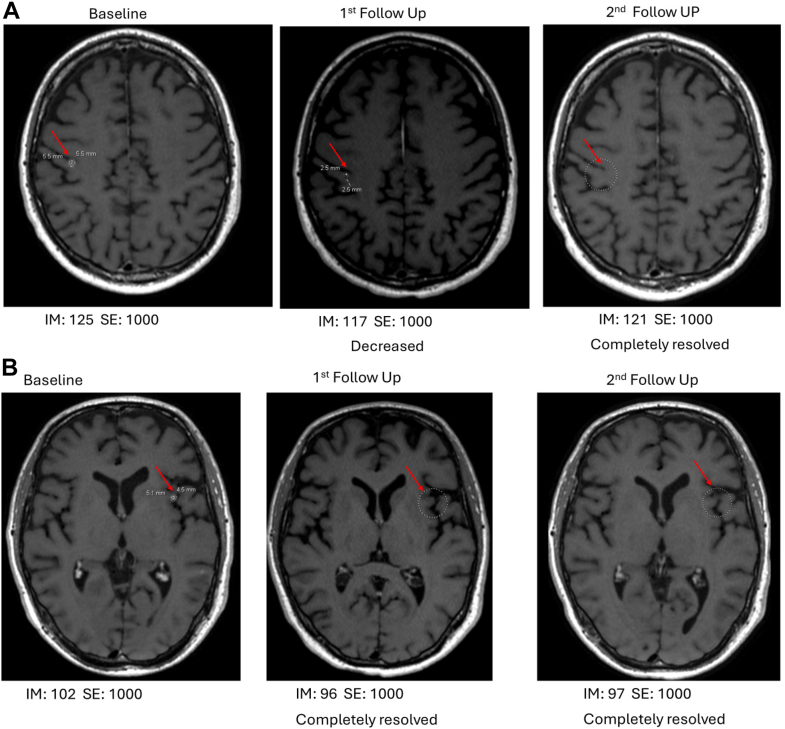
Intracranial response to tarlatamab plus lorlatinib. (*A* and *B*) Complete resolution of a brain metastasis.

Given he was asymptomatic from small albeit numerable brain metastases, a decision was made to initiate tarlatamab first while deferring additional brain radiation. Per institutional protocol, the patient was monitored closely for cytokine release syndrome (CRS) and immune-effector cell–associated neurotoxicity syndrome (ICANS) in the hospital for 24 hours. Signs and symptoms for CRS and ICANS were monitored every 4 hours. Patient’s tarlatamab course was complicated by grade 1 CRS after the first two doses but otherwise therapy was well tolerated with no new or additional adverse events reported. His CRS symptoms were managed by antipyretic and supportive care with resolution of symptoms within 48 hours of onset. After 1 cycle of tarlatamab, PET and brain MRI demonstrated partial responses both extracranially (per clinically report) and intracranially (per modified Response Assessment in Neuro-Oncology—Brain Metastases criteria). This response was sustained and confirmed with repeat CT after 2 cycles of tarlatamab. However, it is difficult to measure the extent of response using Response Evaluation Criteria in Solid Tumors given that target systemic lesions have been previously radiated with no measurable disease in the body (Fig. 2*A–C*). Brain MRI revealed near complete resolution of multiple subcentimeter lesions. There were three lesions measured at 0.6 centimeter, 0.6 centimeter, and 0.5 centimeter with complete resolution of all lesions after 2 cycles of tarlatamab. This finding was confirmed as partial response based on modified Response Assessment in Neuro-Oncology—Brain Metastases (Fig. 3*A* and *B*). Given that the patient has mixed histologies of *ALK*-rearranged adenocarcinoma and small cell carcinoma, he has tolerated lorlatinib well with no notable toxicities reported. Patient continued lorlatinib 100 mg daily while receiving tarlatamab. No drug-drug interaction was noted between lorlatinib and tarlatamab. At the last follow-up of 3 months after initiating tarlatamab treatment, patient continued treatment without toxicities, and both intracranial and systemic responses were sustained. Patient moved to another institution for treatment and was lost to follow-up after this visit.

## Discussion

SCLC transformation from NSCLC harboring actionable oncogenic alterations (AGAs) is a well-characterized clinical phenomenon especially in the era of potent TKI-targeted therapies against driver alterations, including *EGFR* and *ALK*. The SCLC transformation is associated with overall poor clinical outcome and lack of standard-of-care therapies, posing a particular challenge to patients and physicians. Given the nature of mixed histologies and possibly non-overlapping pathway dependence, targeting both AGA-NSCLC and SCLC in the post-transformed setting is a rational strategy.

After small cell transformation, our patient was treated on several regimens for SCLC, including platinum-based chemotherapy and immunotherapy combination, with the continuation of lorlatinib. At the time of further progression, tarlatamab was chosen given tissue DLL3 expression of more than 90% by immunohistochemistry and lack of other standard-of-care treatment options. In the DeLLPhi-301 trial, tarlatamab demonstrated promising efficacy in previously treated patients with SCLC with a response rate of 40% and median PFS of 4.9 months.^11^ This result was further confirmed with DeLLPhi-304. Compared with current Food and Drug Administration–approved second-line treatment for SCLC, tarlatamab was associated with significantly longer OS (13.6 versus 8.3 mo, HR 0.69, 95% CI: 0.47–0.77). In addition, patients with brain metastases were included in the study and derived survival benefit based on prespecified subgroup analysis (OS HR 0.45, 95% CI: 0.31–0.65; PFS HR 0.6, 95% CI: 0.45–0.8).^12^ A recent real-world cohort case series also demonstrated impressive intracranial responses to tarlatamab in patients with numerous untreated, even symptomatic brain metastases.^14^ However, DLL3 positivity was not required to receive treatment nor that has DLL3 score been correlated with efficacy outcomes in the DeLLPhi clinical trials. Patients with small cell transformation were also excluded from the studies, and data for use of tarlatamab in this patient population did not exist before this case report. Yet, DeLLPhi-304 results further solidified previous findings of tarlatamab inducing rapid intracranial response and even in patients with multiple brain metastases.^14^ Our patient had intracranial response after 1 month of lorlatinib and tarlatamab with sustained benefit. This aligns with the current literature findings. Our case demonstrates that the combination of tarlatamab and lorlatinib provides control of systemic disease and response in brain metastases. Notably, the patient had primary progression after a DLL3 CAR T-cell therapy clinical trial before initiating tarlatamab. Possible mechanisms for resistance include antigen heterogeneity, inadequate T-cell potency, and impaired trafficking to the tumor site.^15^ We speculate that bispecific antibody may overcome some of these challenges when given after CAR T-cell therapy with direct engagement of T cells and less T-cell exhaustion.

Our patient has tolerated lorlatinib well with no neurologic or metabolic toxicities. When adding tarlatamab to lorlatinib, the patient did not experience any ICANS or other neurologic toxicities from lorlatinib. This safety signal was reassuring and provides grounds for future investigation. Continuing lorlatinib may contribute to intracranial disease management and provided sustained disease control of the *ALK*-rearranged adenocarcinoma.

In addition, *TP53* and *RB1* alterations are frequently associated with small cell transformation in patients with *EGFR*-mutated NSCLC, and whether it is preexisting and/or potential treatment-emergent resistance clones is a matter of ongoing investigation.^9^^,^^10^^,^^16^^,^^17^ However, the role of *RB1* and *TP53* alterations is less characterized in *ALK*-rearranged NSCLC transformation to SCLC. Our patient developed *RB1* and *PTEN* loss after progressing on several lines of therapies for small cell transformation yet with the TP53 mutation present throughout. Li et al.^5^ reported that in a patient case with small cell transformation after lorlatinib, *RB1* and *TP53* mutations were present since diagnosis and variant allele frequency was increased at the time of transformation. However, other cases have reported transformation along with acquired ALK mutation instead of *RB1* and *TP53* mutations.^6^, ^7^, ^8^ It warrants further investigation to identify possible molecular associations with small cell transformation in patients with *ALK*-rearranged NSCLC.^5^, ^6^, ^7^, ^8^

## Conclusion

Although limited by the short follow-up period after therapy, this case illustrates that tarlatamab is a potential option in *ALK*-rearranged NSCLC with small cell transformation and high DLL3 expression. Importantly, there is no additional toxicity observed when combining tarlatamab with lorlatinib. No additional radiation was needed for either brain or systemic disease at last follow-up. Many questions remain regarding optimal biomarkers for DLL3-targeted therapy selection, such as DLL3 expression level and immune-based biomarkers. Curiously, in this case, the patient did not respond to DLL3 targeting CAR T-cell therapy but subsequently responded to tarlatamab including rapid improvement in intracranial metastases. The concurrent genomic alterations in *ALK*-rearranged NSCLC associated with SCLC transformation also warrant further investigation and validation in a larger patient cohort.

## CRediT Authorship Contribution Statement

**Kaiwen Wang:** Investigation, Data curation, Writing - original draft, Visualization.

**Celyan Altintas Taslic:** Data curation, Writing - review & editing, Visualization.

**Patricia de Groot:** Data curation, Writing - review & editing.

**Mitchell A. Parma:** Data curation, Writing - review & editing.

**Alvaro Guimaraes Paula:** Data curation, Writing - review & editing.

**Melody Caranto:** Data curation.

**Komal Shah:** Supervision, Writing - review & editing, Validation.

**Mukulika Bose:** Data curation.

**Cole Ruoff:** Data curation.

**Loukia G. Karacosta:** Data curation, Visualization.

**Lauren A. Byers:** Writing - review & editing, Supervision.

**Carl M. Gay:** Writing - review & editing, Supervision.

**Jianjun Zhang:** Writing - review & editing.

**John V. Heymach:** Supervision, Writing - review & editing.

**Bingnan Zhang:** Writing - original draft, Supervision, Validation.

## Disclosure

Dr. Wang reports receiving honoraria from MJH Life Sciences and serving on the advisory board with Pfizer and Janssen. Dr. de Groot reports receiving royalties from textbooks from Springer Nature. Dr. Guiamaraes Paula reports receiving honoraria from Pfizer. Dr. Byers reports having consulting/advisory role from AbbVie, Amgen, AstraZeneca, Boehringer Ingelheim, Chugai Pharmaceutical Co., Daiichi Sankyo, Genentech Inc., Jazz Pharmaceuticals, Novartis, and Puma Biotechnology; receiving honoraria from Clinical Care Options and UpToDate; receiving research funding from 10.13039/100002429Amgen, 10.13039/100004325AstraZeneca, and Bristol Myers Squibb; having patents, royalties, and other intellectual property: molecular subtyping of SCLC to predict therapeutic responses (U.S. patent no.: 11,732,306); methods and systems for diagnosis, classification, and treatment of SCLC and other high-grade neuroendocrine carcinomas. Dr. Gay reports having speaking engagement for ACHL, Dava Oncology, IDEOlogy, IDR, Impact Education, MJH, OncLive, PeerView, Physicians’ Education Resource (PER), and Targeted Healthcare; serving on the advisory board/steering committee of Abdera, Amgen, AstraZeneca, BeOne, BioNTech, Boehringer Ingelheim, Daiichi Sankyo, G1 Therapeutics, Jazz Pharmaceuticals, Merck, OncoHost, and Roche/Genentech; providing paid consulting for Axiom, Boehringer Ingelheim, Cantor Fitzgerald, Catalyst, and Pontifax. Dr. J. Zhang reports receiving research funding from Helius, 10.13039/100004331Johnson and Johnson, 10.13039/100004334Merck, 10.13039/100004336Novartis, and Summit, and personal fees from AstraZeneca, Catalyst, GenePlus, Helius, Hengrui, Innovent, Johnson and Johnson, Novartis, Oncohost, Takeda, and Varian, outside the submitted work. Dr. Heymach reports serving on the advisory committees of AstraZeneca, AbbVie, AnHeart, Boehringer Ingelheim, Bayer, BioAtla, BioNTech, Bristol Myers Squibb, Dizal, Ellipsis, EMD Serono, Genentech, GlaxoSmithKline, Hengrui Therapeutics, Janssen Global Services, Eli Lilly, ModeX Therapeutics, Novartis, Pfizer, Remunity, Sanofi, Spectrum, Takeda, Leads Biolabs, and Regeneron; receiving research support from AstraZeneca, Spectrum, Boehringer Ingelheim, Taiho, Bristol Myers Squibb, and Takeda; and receiving royalties and licensing fees from Spectrum. Dr. B. Zhang reports receiving advisory board fees from Abdera Therapeutics, Daiichi Sankyo, and OncoHost; receiving research support from 10.13039/100007038Lung Cancer Research Foundation; and receiving honoraria from IDEOlogy Health. The remaining authors declare no conflict of interest.

## References

[1] Tan A.C., Tan D.S.W. (2022). Targeted therapies for lung cancer patients with oncogenic driver molecular alterations. J Clin Oncol.

[2] Mok T., Camidge D.R., Gadgeel S.M. (2020). Updated overall survival and final progression-free survival data for patients with treatment-naive advanced ALK-positive non-small-cell lung cancer in the ALEX study. Ann Oncol.

[3] Camidge D.R., Kim H.R., Ahn M.J. (2020). Brigatinib versus crizotinib in advanced ALK inhibitor-naive ALK-positive non-small cell lung cancer: second interim analysis of the phase III ALTA-1L trial. J Clin Oncol.

[4] Solomon B.J., Liu G., Felip E. (2024). Lorlatinib versus crizotinib in patients with advanced ALK-positive non-small cell lung cancer: 5-year outcomes from the Phase III CROWN study. J Clin Oncol.

[5] Li H., Song T., Xu X. (2023). ALK-positive adenocarcinoma after acquired resistance to lorlatinib and transformation to SCLC: a case report. JTO Clin Res Rep.

[6] Majeed U., Li S., Seegobin K. (2023). First report of management of sequential small cell transformation and ALK I1171T mutation as resistance mechanisms in a patient with ALK-EML4 fused non-small cell lung adenocarcinoma with a novel combination of temozolomide and lorlatinib: a case report. JTO Clin Res Rep.

[7] Coleman N., Wotherspoon A., Yousaf N., Popat S. (2019). Transformation to neuroendocrine carcinoma as a resistance mechanism to lorlatinib. Lung Cancer.

[8] Lingling X., Maoxi C., Wei Y., Jieting Z., Yuanyuan Y., Ning X. (2023). Transformation of NSCLC to SCLC harboring EML4-ALK fusion with V1180L mutation after alectinib resistance and response to lorlatinib: a case report and literature review. Lung Cancer.

[9] Zhang B., Lewis W., Stewart C.A. (2023). Brief report: comprehensive Clinicogenomic profiling of small cell transformation from EGFR-mutant NSCLC informs potential therapeutic targets. JTO Clin Res Rep.

[10] Marcoux N., Gettinger S.N., O’Kane G. (2019). EGFR-mutant adenocarcinomas that transform to small-cell lung cancer and other neuroendocrine carcinomas: clinical outcomes. J Clin Oncol.

[11] Ahn M.J., Cho B.C., Felip E. (2023). Tarlatamab for patients with previously treated small-cell lung cancer. N Engl J Med.

[12] Mountzios G., Sun L., Cho B.C. (2025). Tarlatamab in small-cell lung cancer after platinum-based chemotherapy. N Engl J Med.

[13] Karacosta L.G., Pancirer D., Preiss J.S. (2023). Phenotyping EMT and MET cellular states in lung cancer patient liquid biopsies at a personalized level using mass cytometry. Sci Rep.

[14] Zhang B., Shah K.B., Parma M. (2025). Rapid intracranial response with tarlatamab in patients with untreated brain metastases from SCLC-A real-world case series: case report. J Thorac Oncol.

[15] Labanieh L., Mackall C.L. (2023). CAR immune cells: design principles, resistance and the next generation. Nature.

[16] Niederst M.J., Sequist L.V., Poirier J.T. (2015). RB loss in resistant EGFR mutant lung adenocarcinomas that transform to small-cell lung cancer. Nat Commun.

[17] Oser M.G., Niederst M.J., Sequist L.V., Engelman J.A. (2015). Transformation from non-small-cell lung cancer to small-cell lung cancer: molecular drivers and cells of origin. Lancet Oncol.

